# Impaired α-Granule Secretion Dominates Longitudinal Agonist-Induced Platelet Dysfunction in Gaucher Disease

**DOI:** 10.3390/ijms27188263

**Published:** 2026-09-16

**Authors:** Shoshana Revel-Vilk, Ari Zimran, Tama Dinur, Dafna Frydman, Elena Shulman, Emmanuel Benayoun, Eti Broide, Mira Naamad, Nechama Koren, Michal Saltsman

**Affiliations:** 1 Gaucher Unit, The Eisenberg R&D Authority, Shaare Zedek Medical Center, Jerusalem 9103102, Israel; azimran@gmail.com (A.Z.);; 2 Faculty of Medicine, Hebrew University of Jerusalem, Jerusalem 9112002, Israel; 3 Agyany Pharma Ltd., Jerusalem 9695614, Israel; 4 Flow Cytometry Unit, Shaare Zedek Medical Center, Jerusalem 9103102, Israel

**Keywords:** Gaucher disease, platelet activation, agonist-induced platelet function, α-granule secretion, CD62P, P-selectin, flow cytometry

## Abstract

Bleeding in Gaucher disease (GD) is not fully explained by thrombocytopenia and coagulation disorders. Previous studies have shown impaired agonist-induced cluster of differentiation (CD) 62P (CD62P/P-selectin) responses, but their persistence over time is unknown. This retrospective longitudinal observational study characterized platelet activation and secretion responses over time and factors associated with persistent abnormalities. Whole-blood flow cytometry studies from 333 patients with GD with at least two assessments were analyzed. Platelet activation complex-1 (PAC1), CD62P, and CD63 responses were categorized longitudinally. Patients contributed 949 visits over a median follow-up of 2.3 years. Persistent CD62P abnormality was most frequent (92/333, 27.6%), compared with PAC1 (45/329, 13.7%) and CD63 (9/328, 2.7%). PAC1 abnormalities were more often dynamic, whereas CD62P abnormalities were frequent and persistent, most often involving thrombin receptor-activating peptide 6 (TRAP-6) and cross-linked collagen-related peptide (CRP-XL). Lower platelet count was independently associated with persistent CD62P abnormality, although approximately half of affected patients had platelet counts ≥150 × 10^9^/L. Treatment throughout follow-up was associated with lower odds of persistent CD62P and CD63 abnormalities. Impaired CD62P expression was the predominant persistent abnormality, consistent with preferential impairment of α-granule secretion. Platelet function changed over time, supporting reassessment in patients with bleeding manifestations and before procedures when previous testing is remote or clinical status has changed.

## 1. Introduction

Gaucher disease (GD) is an inherited lysosomal storage disorder caused by deficient glucocerebrosidase activity, leading to accumulation of glucosylceramide and related sphingolipids. This accumulation occurs predominantly within cells of the monocyte–macrophage system, resulting in infiltration of the spleen, liver, and bone marrow and contributing to the major systemic manifestations of the disease [1]. The hematologic manifestations of GD include anemia, thrombocytopenia, splenomegaly, and bleeding [2]. Thrombocytopenia in GD is multifactorial, resulting primarily from hypersplenism associated with splenomegaly and impaired megakaryopoiesis related to bone-marrow infiltration by Gaucher cells [3]. Although thrombocytopenia is clinically important, bleeding severity in GD is often incompletely explained by platelet count alone, suggesting that qualitative platelet dysfunction contributes to the hemostatic phenotype. Abnormal platelet aggregation, adhesion, and agonist-induced activation have been described in patients with GD, including in patients without severe thrombocytopenia [4,5].

Platelet hemostatic function depends on coordinated agonist-induced signaling, αIIbβ3 integrin activation, and granule secretion [6]. Flow cytometry enables pathway-specific assessment of these processes in whole blood. Platelet activation complex-1 (PAC1) binding reflects activation of αIIbβ3 integrin, cluster of differentiation (CD) 62P (CD62P/P-selectin) surface expression reflects α-granule secretion, and CD63 surface expression reflects dense-granule and lysosomal secretion [7,8,9].

Our previous cross-sectional work showed that GD-associated platelet dysfunction is not uniform across platelet pathways, with reduced agonist-induced CD62P responses emerging as a prominent abnormality [5]. Higher glucosylsphingosine (lyso-Gb1), a biomarker of GD burden [10], was associated with more severe platelet dysfunction. However, cross-sectional data cannot determine whether these abnormalities are stable patient-level traits, transient findings, or consequences of changing disease state, treatment exposure, or platelet count over time. We therefore performed a longitudinal platelet flow cytometry study in patients with GD who underwent repeated platelet function testing. We hypothesized that persistent platelet dysfunction in GD would be dominated by impaired α-granule secretion, reflected in persistent abnormal agonist-induced CD62P expression during follow-up. We further evaluated the longitudinal patterns of PAC1, CD62P, and CD63 responses and clinical factors associated with persistent abnormalities. We found that impaired CD62P expression was the predominant persistent abnormality, consistent with preferential impairment of α-granule secretion. We observed persistent platelet dysfunction also in patients with normal platelet counts and in those receiving GD-specific therapy, although GD-specific treatment was associated with a lower likelihood of persistent platelet dysfunction.

## 2. Results

### 2.1. Cohort Characteristics

The study included 333 patients with GD, representing a broad age range, with most patients having an intact spleen and platelet counts within or near the normal range, and a median of 3 platelet function assessments over a median follow-up of 2.3 years (Table 1). Most patients were classified as type 1 GD (325, 97.6%), while 8 (2.4%) had type 3 GD; no patients had type 2 GD.

### 2.2. Longitudinal Platelet Function

**Figure 1:**
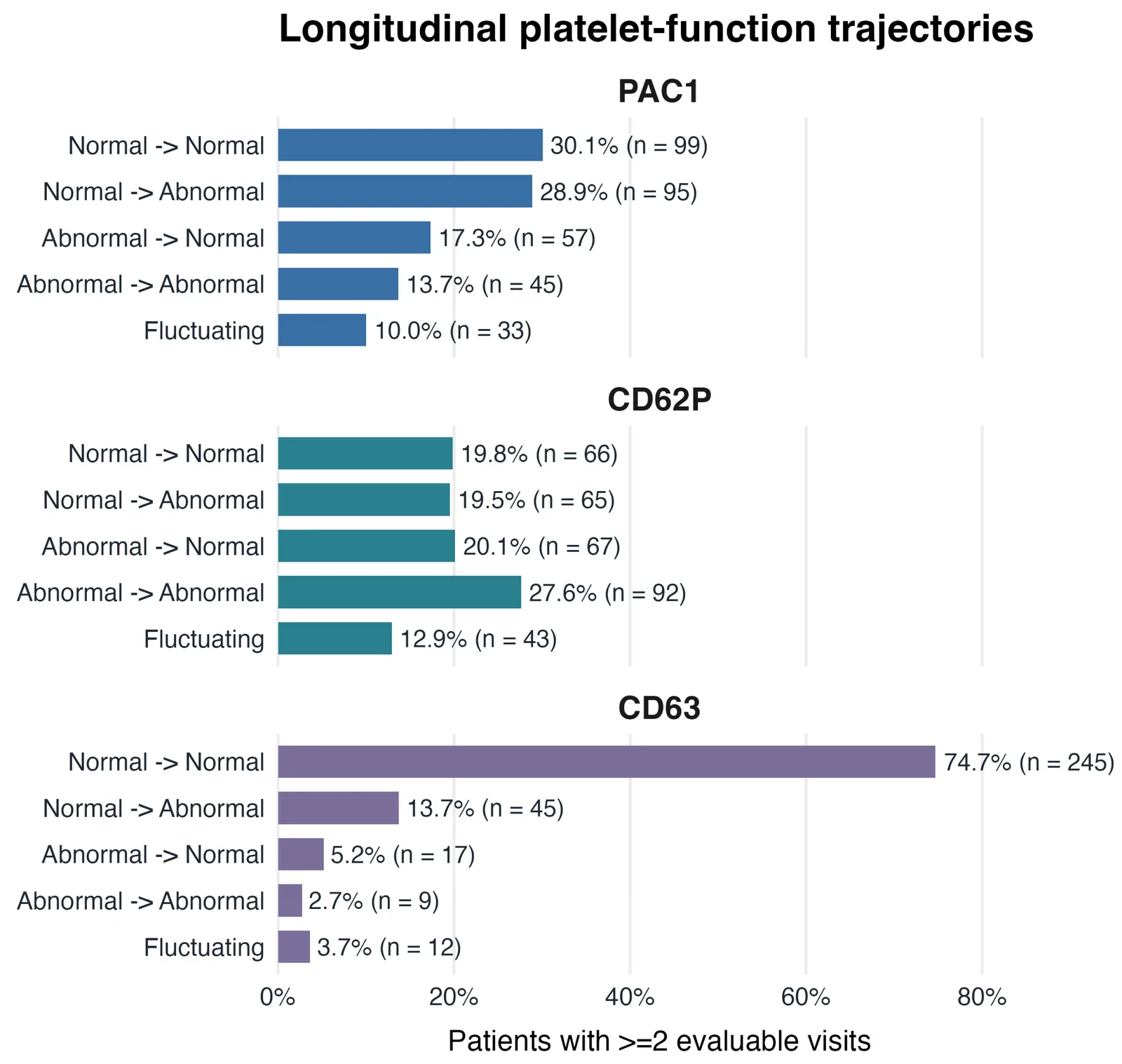
Longitudinal trajectories of platelet function responses. Patient-level trajectories of agonist-induced PAC1 binding, CD62P expression, and CD63 expression across repeated flow cytometry assessments. Arrows indicate the direction of change in platelet-function status between assessments. PAC1, platelet activation complex-1; CD, cluster of differentiation.

Longitudinal patterns differed among the three activation-dependent responses (Figure 1). PAC1 abnormalities were common but were more often dynamic than persistent. Abnormal agonist-induced CD62P expression was more frequent and more often persistent than abnormalities in PAC1 binding or CD63 expression. CD63 abnormalities were uncommon, and most patients maintained normal CD63 expression throughout follow-up (Table 1, Figure 1).

**Figure 2:**
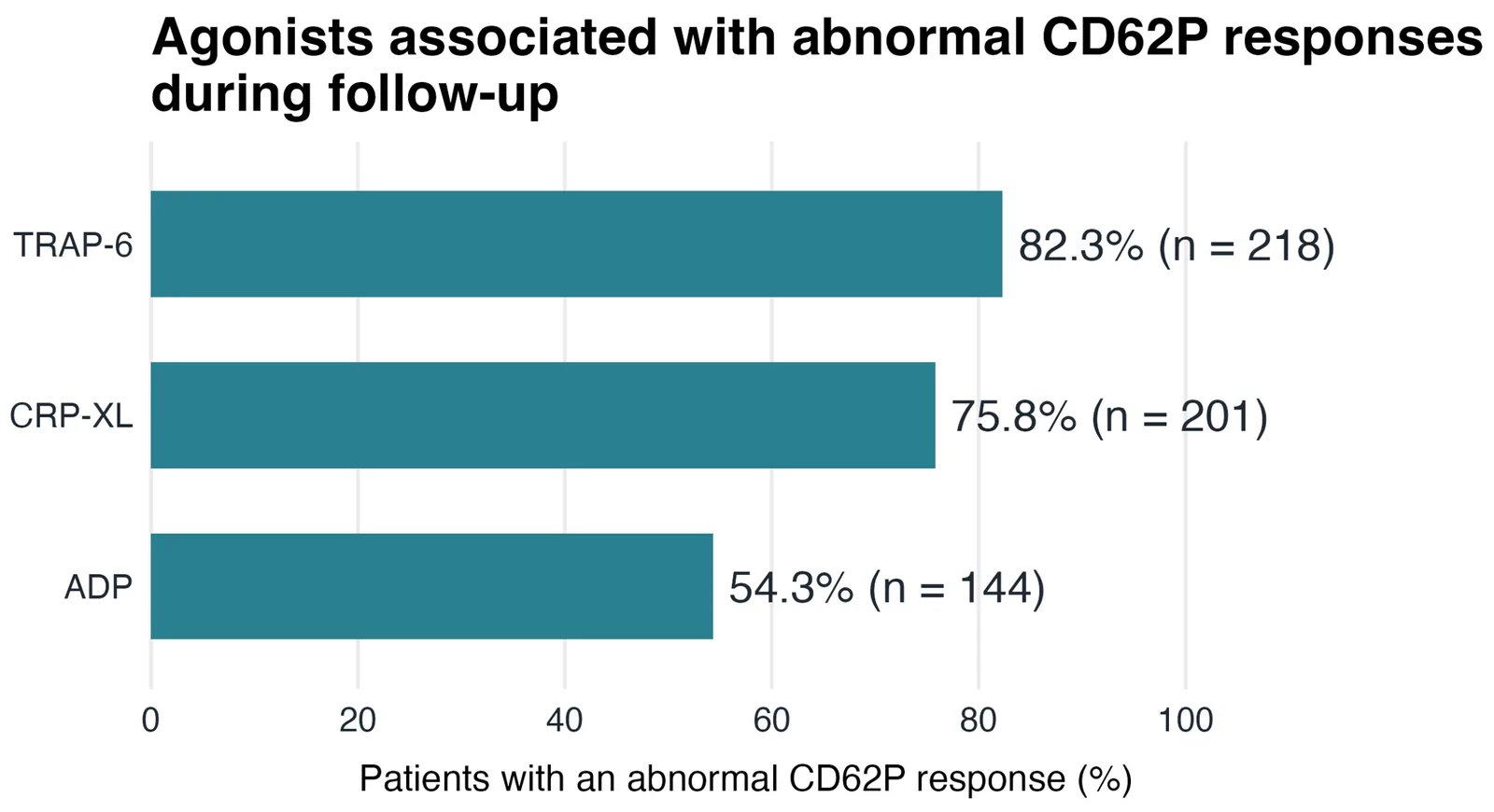
CD62P agonist-response patterns. Frequency of abnormal CD62P responses by agonist condition among patients with any CD62P abnormality during follow-up. ADP, adenosine diphosphate; CRP-XL, cross-linked collagen-related peptide; TRAP-6, thrombin receptor-activating peptide 6.

Among patients with abnormal CD62P responses during follow-up, abnormalities were mainly following thrombin receptor-activating peptide 6 (TRAP-6) and cross-linked collagen-related peptide (CRP-XL) stimulation (Figure 2).

### 2.3. Factors Associated with Persistent Platelet Function Abnormalities

In univariable analyses, longer follow-up duration and treatment throughout follow-up were associated with lower odds of persistent abnormalities across all three platelet responses (Table 2). Lower platelet count was associated with higher odds of persistent CD62P abnormality. Age, lyso-Gb1, genotype classification, and splenectomy status were not significantly associated with persistent PAC1, CD62P, or CD63 abnormalities in univariable analyses (Table 2).

In multivariable analyses, longer follow-up duration remained associated with lower odds of persistent abnormalities across all three platelet responses. Treatment throughout follow-up was associated with lower odds of persistent CD62P and CD63 abnormalities, while the association with persistent PAC1 abnormality did not remain statistically significant. Lower platelet count remained independently associated with higher odds of persistent CD62P abnormality. Although platelet counts were lower in patients with persistent CD62P abnormality, the distributions overlapped substantially: median platelet counts were 151.5 × 10^9^/L (IQR, 101.8–199) versus 178.5 × 10^9^/L (IQR, 141–225), a difference of 27 × 10^9^/L. Moreover, 47 of 92 (51.1%) patients with persistent CD62P abnormality had platelet counts ≥150 × 10^9^/L.

## 3. Discussion

In this longitudinal study of patients with GD undergoing repeated platelet function testing, abnormalities differed among the three activation-dependent responses. Impaired agonist-induced CD62P expression was the most frequent and most persistent abnormality, whereas PAC1 abnormalities were more often dynamic, and persistent CD63 abnormalities were uncommon. Treatment throughout follow-up was associated with lower odds of persistent CD62P and CD63 abnormalities, and lower platelet count was independently associated with persistent CD62P abnormality.

These findings extend our previous cross-sectional study, in which reduced platelet reactivity was common in GD and impaired P-selectin reactivity was associated with bleeding manifestations [3]. The predominance of persistent CD62P abnormalities over PAC1 and CD63 abnormalities is consistent with preferential impairment of α-granule secretion rather than a generalized defect of platelet activation or secretion [5,6]. This interpretation is further supported by CD62P abnormalities following both TRAP-6 and CRP-XL, which signal through distinct receptors, suggesting involvement of downstream mechanisms shared across activation pathways. Our previous finding that serum brain-derived neurotrophic factor (BDNF), an α-granule protein, correlated with stimulated CD62P expression and was lower in patients with increased bleeding manifestations further supports α-granule involvement in GD [11]. A potential mechanism may involve α-synuclein, which is highly expressed in platelets and has been shown experimentally to inhibit Ca^2+^-dependent α-granule release without similarly affecting dense- or lysosomal-granule release [12]. Given the relationship between *GBA1* and α-synuclein biology [13], this warrants further investigation. Studies assessing α-granule content and release could help distinguish altered granule content from impaired secretion [14,15]. Future prospective studies of platelet signaling pathways and proteins may provide further insight into the mechanisms underlying the persistent CD62P abnormality. 

Lower platelet count was independently associated with persistent CD62P abnormality; however, the absolute difference in platelet counts was modest, with substantial overlap between groups. Importantly, approximately half of patients with persistent CD62P abnormality had platelet counts ≥150 × 10^9^/L. Thus, persistent platelet dysfunction was not confined to patients with thrombocytopenia, consistent with previous observations that qualitative platelet abnormalities in GD may occur despite relatively preserved platelet counts [2,3].

Treatment throughout follow-up was associated with lower odds of persistent CD62P and CD63 abnormalities, whereas the association with PAC1 abnormality did not remain significant after adjustment. Previous small studies have shown that platelet aggregation may improve or normalize during enzyme replacement therapy (ERT), although abnormalities persisted in some patients [4,16,17]. Other aspects of platelet function may be less responsive to treatment: platelet adhesion under high-shear conditions was not corrected by ERT [18], and Komninaka et al. reported frequent abnormal PFA-100 closure times despite long-term ERT [19]. Together, these findings suggest that GD-specific treatment may improve platelet function without uniformly correcting the underlying dysfunction.

Flow cytometry provides additional information beyond conventional platelet aggregation studies. Whereas light transmission aggregometry measures the overall aggregation response to an agonist, flow cytometry allows assessment of specific components of platelet activation and secretion [5,6]. In particular, agonist-induced CD62P expression provides a specific measure of α-granule secretion, which may be abnormal despite preserved aggregation [20]. Thus, the predominance of persistent CD62P abnormalities in our study identifies a component of platelet dysfunction in GD that may not be apparent from conventional aggregometry alone.

The dynamic changes observed in platelet function also have practical implications for following patients with GD. Current recommendations support regular assessment of bleeding manifestations and hemostatic evaluation when clinically indicated, particularly before invasive procedures [21]. Our findings suggest that a previous platelet function result may not reliably reflect current platelet function, as abnormalities may develop or resolve over time. Repeat platelet function assessment should therefore be considered in patients with a bleeding tendency and before procedures associated with bleeding risk, particularly when previous testing is remote, or the clinical or treatment status has changed. Current recommendations for GD include antifibrinolytic therapy and platelet transfusion when clinically indicated for peri-procedural hemostatic support [3,22]. Desmopressin has shown hemostatic benefit in some platelet function disorders [23], and its potential role in the platelet secretion phenotype observed in GD warrants further investigation. Although GD-specific treatment was associated with a lower likelihood of persistent CD62P abnormalities, GD-specific therapy requires time to improve hematologic manifestations and therefore would not be expected to provide immediate correction of platelet dysfunction before a planned invasive procedure [21,22]. Optimization of GD-specific therapy may nevertheless be relevant in patients with ongoing bleeding manifestations.

This study has several limitations. Its retrospective design resulted in variation in the number and timing of platelet function assessments and duration of follow-up. Importantly, persistence was defined as an abnormal response at every evaluable assessment; therefore, patients with longer follow-up and more assessments had more opportunities to have a normal result and not meet the definition of persistent abnormality. This may at least partly explain the inverse association between follow-up duration and persistent abnormalities. Platelet flow cytometry is also sensitive to preanalytical and technical factors, although testing was performed using a standardized laboratory protocol and technically uninterpretable stimulated responses were excluded according to predefined quality-control criteria. The assay identifies abnormalities in agonist-induced platelet responses but cannot determine the underlying signaling or granule defect. Finally, bleeding outcomes were not evaluated longitudinally, limiting assessment of the clinical significance of persistent or changing platelet function abnormalities.

## 4. Materials and Methods

### 4.1. Study Design and Population

This was a longitudinal observational study of patients with GD who underwent repeated platelet function testing by whole-blood flow cytometry. Patients were eligible for the longitudinal analysis if they had at least two platelet function assessments. Patient-level variables included age, sex, genotype-based mild disease classification, splenectomy status, treatment exposure, platelet count, and lyso-Gb1. Genotype was classified as mild for N370S homozygous (traditional nomenclature) [p.Asn409Ser homozygous] or N370S/R496H (traditional nomenclature) [p.Asn409Ser/p.Arg535His], with all other genotypes classified as non-mild. This genotype-based classification served as a surrogate for disease severity. Lyso-Gb1 was measured in dried blood spots by liquid chromatography–tandem mass spectrometry (LC-MS/MS) at CENTOGENE(Rostock, Germany), as previously described [24]. For repeated laboratory measurements, platelet count and lyso-Gb1 were summarized at the patient level using the median of available observations. Gaucher disease-specific treatment included ERT and substrate reduction therapy (SRT). Treatment exposure during follow-up was classified as never treated, treated throughout follow-up, or mixed treatment. Patients were classified as never treated if they were untreated at all assessments, treated throughout if they were receiving treatment at all assessments, and mixed for all other treatment patterns during follow-up.

### 4.2. Platelet Function Testing by Flow Cytometry

Platelet function testing was performed in acid-citrate-dextrose anticoagulated whole blood using the flow-cytometry assay described previously [3]. In brief, the first 1 mL of blood was discarded, and samples were left at room temperature for 30 min before testing to reduce pre-analytical platelet activation. Platelets were identified using CD42b, and activation-dependent surface responses were expressed as the percentage of positive platelets within the platelet population. Mean fluorescence intensity (MFI) was not used as an outcome. Laboratory-specific reference ranges were established using healthy control samples tested with the same standardized flow-cytometry protocol [8,25]. The same predefined reference ranges were applied throughout the longitudinal study, and healthy controls were tested periodically to verify that assay responses remained consistent with these ranges, as part of routine laboratory quality control. The longitudinal analysis focused on agonist-induced PAC1 binding, CD62P/P-selectin expression, and CD63 expression after high-dose adenosine diphosphate (ADP) (20 μM) (moLab GmbH, Unna, Germany), high-dose TRAP-6 (20 μM) (BACHEM, Dubendorf, Switzerland), and CRP-XL (0.25 μg/mL) (Cambcol Laboratories, Ely, UK). These agonist concentrations were part of the standardized flow cytometry protocol established in our previous study and were kept unchanged throughout the study period to ensure comparability of longitudinal assessments. The platelet function readouts were interpreted as follows: PAC1 binding reflected agonist-induced αIIbβ3 integrin activation; CD62P/P-selectin surface expression reflected α-granule secretion; and CD63 surface expression reflected dense-granule/lysosome-associated secretion [3]. Platelets were identified using phycoerythrin–cyanine 5 (PE-Cy™5) mouse anti-human CD42b (BD Biosciences, San Jose, CA, USA; catalog no. 551141; working concentration, 1 µg/mL). Platelet activation and secretion were assessed using fluorescein isothiocyanate (FITC) anti-human PAC-1 (BD Biosciences, San Jose, CA, USA; catalog no. 340507; 5 µg/mL), allophycocyanin (APC) anti-human CD62P/P-selectin (BioLegend, San Diego, CA, USA; catalog no. 304910; 3.6 µg/mL), and Pacific Blue™ anti-human CD63 (BioLegend, San Diego, CA, USA; catalog no. 353012; 16 µg/mL). Samples were analyzed using a BD FACSCanto™ II flow cytometer (BD Biosciences, San Jose, CA, USA).

### 4.3. Longitudinal Trajectory Definitions

For each platelet function response, individual agonist-response results were first classified as abnormal or normal according to the laboratory-specific reference ranges described above. At each visit, the response was classified as abnormal if any evaluable agonist condition for that response was abnormal, normal if all required agonist conditions were evaluable and normal, and indeterminate otherwise. Stimulated PAC1, CD62P, and CD63 responses with ≤5% positive platelets were considered technically uninterpretable based on the laboratory’s established criteria for technical assay failure and were excluded before visit-level classification. These values were classified as neither normal nor abnormal.

Patients with at least two evaluable visits for a given platelet function response were assigned to one of five longitudinal trajectory categories based on the full sequence of evaluable visit-level results: normal throughout, normal → abnormal, abnormal → normal, abnormal throughout, or fluctuating. Persistent abnormality was defined as an abnormal response at every evaluable assessment for that platelet function response. Fluctuating was assigned when the evaluable sequence contained both normal and abnormal results but did not meet the normal → abnormal or abnormal → normal definitions, including sequences with intervening reversals or identical first and last statuses with an opposite status at another evaluable visit.

### 4.4. Statistical Analysis

Continuous variables are summarized as medians with IQRs, and categorical variables are summarized as counts and percentages. Persistent abnormality rates were calculated using response-specific evaluable denominators. Univariable logistic regression was used to estimate the odds ratio (OR), 95% confidence interval (CI), and *p*-value for each candidate predictor in relation to persistent abnormality in PAC1 binding, agonist-induced CD62P expression, and CD63 expression. Candidate predictors were age, follow-up duration, platelet count, lyso-Gb1, sex, genotype-based mild disease classification, splenectomy status, and treatment group. Variables with a *p*-value ≤ 0.10 in the corresponding univariable logistic regression were included in the response-specific multivariable logistic regression model, provided that the variable was estimable. Multicollinearity among candidate predictors was assessed before model fitting. Results are reported as ORs and adjusted ORs with 95% CIs and *p*-values. A two-sided *p*-value < 0.05 was considered statistically significant. Statistical analyses were performed using R version 4.5.2 (R Foundation for Statistical Computing, Vienna, Austria).

## 5. Conclusions

Longitudinal flow-cytometric platelet function testing in GD demonstrates that impaired agonist-induced CD62P expression is the most frequent and persistent abnormality, consistent with preferential impairment of α-granule secretion. By assessing specific platelet activation and secretion pathways, flow cytometry identifies a component of platelet dysfunction that is not directly assessed by conventional aggregation testing. Platelet function also changed over time, with abnormalities developing or resolving during follow-up, and persistent dysfunction was not confined to patients with thrombocytopenia. These findings support reassessment of platelet function in patients with bleeding manifestations and before procedures when previous testing is remote or clinical status has changed.

## Figures and Tables

**Table 1:** Cohort characteristics.

Characteristic	Overall	Persistent Abnormal		
		PAC1	CD62P	CD63
Patients	333	45	92	9
Evaluable visits	949	109	237	23
Visits per patient, median (IQR; range)	3 (2–3); 2–7	2 (2–3); 2–6	2 (2–3); 2–6	2 (2–3); 2–4
Follow-up duration, years, median (IQR)	2.3 (1.4–3.3)	2 (0.9–2.7)	2 (1.1–2.9)	1.8 (1–1.9)
Age at first visit, years, median (IQR)	42.1 (25.3–57.2)	43 (29–62.8)	40.4 (25.3–55.6)	52.3 (28.1–62.8)
Sex, female, n (%)	181 (54.4%)	19 (42.2%)	44 (47.8%)	3 (33.3%)
Mild genotype, n (%)	211 (63.4%)	33 (73.3%)	60 (65.2%)	8 (88.9%)
Splenectomy, n (%)	47 (14.1%)	7 (15.6%)	9 (9.8%)	0 (0%)
Gaucher disease—treatment				
Never treated, n (%)	78 (23.4%)	14 (31.1%)	30 (32.6%)	6 (66.7%)
Treated throughout follow-up, n (%)	213 (64%)	22 (48.9%)	47 (51.1%)	2 (22.2%)
Mixed treatment, n (%)	42 (12.6%)	9 (20%)	15 (16.3%)	1 (11.1%)
Platelet count, ×10^9^/L, median (IQR)	172.5 (134–214.5)	170 (123–205)	151.5 (101.8–199)	150 (58–182.5)
Lyso-Gb1, ng/mL, median (IQR)	93.5 (41–187)	59.4 (32.6–152.5)	107.5 (35.6–226.1)	48.6 (37.4–234)

IQR, interquartile range; lyso-Gb1, glucosylsphingosine; PAC1, platelet activation complex-1; CD, cluster of differentiation Mild genotype: N370S homozygous (traditional nomenclature) [p.Asn409Ser homozygous] or N370S/R496H (traditional nomenclature) [p.Asn409Ser/p.Arg535His]. Gaucher disease treatment: enzyme replacement therapy (ERT) and substrate reduction therapy (SRT).

**Table 2:** Univariable and multivariable analyses of persistent platelet-function abnormalities.

Predictor	Univariable OR (95% CI)	*p*-Value	Adjusted OR (95% CI)	*p*-Value
Agonist-induced PAC1 expression				
Age, per year	1.01 (0.99–1.02)	0.353	—	—
Follow-up duration, per year	0.66 (0.48–0.89)	0.007	0.67 (0.48–0.89)	0.007
Platelet count, per 10 × 10^9^/L	0.99 (0.94–1.03)	0.589	—	—
Lyso-Gb1, per unit	1.00 (1.00–1.00)	0.349	—	
Male sex (ref: female)	1.87 (0.97–3.65)	0.062	1.92 (1–3.74)	0.05
Non-mild genotype (ref: mild)	0.59 (0.28–1.17)	0.144	—	—
Splenectomy (ref: intact spleen)	1.12 (0.44–2.56)	0.793	—	—
Treated throughout follow-up (ref: never treated)	0.45 (0.21–0.99)	0.044	0.53 (0.25–1.14)	0.096
Agonist-induced CD62P expression				
Age, per year	0.99 (0.98–1.01)	0.384	—	—
Follow-up duration, per year	0.78 (0.62–0.95)	0.026	0.78 (0.62–0.97)	0.026
Platelet count, per 10 × 10^9^/L	0.96 (0.92–0.99)	0.024	0.96 (0.92–0.99)	0.024
Lyso-Gb1, per unit	1.00 (1.00–1.00)	0.358	—	—
Male sex (ref: female)	1.44 (0.89–2.33)	0.140	—	—
Non-mild genotype (ref: mild)	0.89 (0.54–1.47)	0.664	—	—
Splenectomy (ref: intact spleen)	0.58 (0.25–1.20)	0.165	—	—
Treated throughout follow-up (ref: never treated)	0.53 (0.3–0.96)	0.033	0.53 (0.30–0.96)	0.033
Agonist-induced CD63 expression				
Age, per year	1.01 (0.98–1.05)	0.564	—	—
Follow-up duration, per year	0.48 (0.23–0.91)	0.035	0.5 (0.24–0.95)	0.048
Platelet count, per 10 × 10^9^/L	0.90 (0.80–1.01)	0.089	0.95 (0.84–1.05)	0.35
Lyso-Gb1, per unit	1.00 (0.99–1.00)	0.808	—	—
Male sex (ref: female)	2.52 (0.65–12.13)	0.196	—	—
Non-mild genotype (ref: mild)	0.21 (0.01–1.18)	0.147	—	—
Splenectomy (ref: intact spleen)	Not estimable	—	—	—
Treated throughout follow-up (ref: never treated)	0.13 (0.02–0.58)	0.014	0.15 (0.02–0.73)	0.028

CI, confidence interval; OR, odds ratio; IQR, interquartile range; lyso-Gb1, glucosylsphingosine; PAC1, platelet activation complex-1; CD, cluster of differentiation.

## Data Availability

The data presented in this study are available on request from the corresponding author due to ethical and privacy issues. Access to deidentified data may be considered upon reasonable request, subject to institutional approval and in compliance with ethical and privacy regulations.
